# Desempeño de las pruebas combinadas de IgM e IgG rápidas en la vigilancia ocupacional de COVID-19 en empresas colombianas

**DOI:** 10.7705/biomedica.5829

**Published:** 2020-11-15

**Authors:** Álvaro J. Idrovo, José Moreno-Montoya, Carlos E. Pinzón-Flórez

**Affiliations:** 1 Departamento de Salud Pública, Escuela de Medicina, Universidad Industrial de Santander, Bucaramanga, Colombia Universidad Industrial de Santander Departamento de Salud Pública Universidad Industrial de Santander Bucaramanga Colombia; 2 Subdirección de Estudios Clínicos, Fundación Santa Fe de Bogotá, Bogotá D.C, Colombia Fundación Santa Fe de Bogotá BogotáD.C Colombia; 3 Subgerencia de Medicamentos, EPS Sanitas, Bogotá D.C, Colombia EPS Sanitas BogotáD.C Colombia

**Keywords:** infecciones por coronavirus, epidemias, inmunidad, salud laboral, Coronavirus infections, epidemics, immunity, occupational health

## Abstract

**Introducción.:**

Las pruebas combinadas de IgM e IgG rápidas pueden tener un papel importante en la vigilancia de la COVID-19 y en su diagnóstico, así como en la evaluación de la respuesta inmunológica y la verificación de los avances hacia la inmunidad de rebaño.

**Objetivo.:**

Evaluar el desempeño de las pruebas rápidas de anticuerpos en la vigilancia ocupacional de la COVID-19 en un grupo de empresas colombianas.

**Materiales y métodos.:**

Se usaron datos de la vigilancia ocupacional de empresas que hicieron pruebas serológicas periódicas a todo el personal desde finales de abril hasta comienzos de julio de 2020. Los trabajadores laboraban en grupos pequeños ("burbujas sociales") para evitar brotes y optimizar la vigilancia. La sensibilidad se estimó como si el muestreo respondiera a un diseño prospectivo. Se describieron, asimismo, los cambios en las pruebas serológicas por medio de rondas periódicas.

**Resultados.:**

Se obtuvieron datos de 4.740 trabajadores, de los cuales solo 23 eran sintomáticos. En ellos se evidenciaron cambios de IgM(-)/IgG(-) a IgM(+), y luego a IgM(+)/ IgG(+) e IgG(+). La sensibilidad fue de 40,94 % para las IgM(+) y 47,95 % para las IgM(+)/ IgG(+), lo que implica que se pudo detectar un poco menos de la mitad de los casos.

**Conclusión.:**

Las pruebas rápidas de anticuerpos tienen un papel en el proceso diagnóstico de la infección y deben evaluarse teniendo en cuenta el momento de la epidemia, el tipo de prueba comprada y las poblaciones de riesgo, dado que sus resultados dependen del número de contagios y de casos. En el contexto de la presente crisis sanitaria pueden optimizarse si se organizan los trabajadores en "burbujas sociales".

## Abstract (en)

**Introduction:**

Rapid IgM-IgG combined antibody tests can play an important role in the COVID-19 surveillance by supporting the diagnosis of infection, assessing the immune response, and verifying the progress towards herd immunity.

**Objective:**

To evaluate the performance of rapid IgM-IgG combined antibody tests in COVID-19 occupational surveillance in a group of Colombian enterprises.

**Materials and methods:**

We used the occupational surveillance data from companies that had performed periodic serological tests on all personnel from the end of April to the beginning of July, 2020. Workers were organized in small groups ("social bubbles") to prevent outbreaks and optimize surveillance. The sensitivity was estimated as if the sampling had a prospective design. We describe here the changes in serological testing through periodic rounds.

**Results:**

Data were obtained from 4,740 workers, of whom only 23 were symptomatic showing changes from IgM(-)/IgG(-) to IgM(+) and then to IgM(+)/IgG(+) and IgG(+). The sensitivity was 40.94% for IgM(+) and 47.95% for IgM(+)/IgG(+). This implies that a little less than half of the cases can be detected.

**Conclusion:**

Antibody rapid tests have a role in the diagnostic process of infection and they must be evaluated taking into account the moment of the epidemic, the type of test purchased, and the populations at risk since their results depend on the number of infections and cases. In the context of a health crisis, they can be optimized by organizing workers into "social bubbles".

La pandemia de COVID-19 ha resultado ser un desafío sin precedentes como crisis sanitaria global. Más allá de la infección, la enfermedad y la muerte asociadas con el SARS-CoV2, con la pandemia se han evidenciado múltiples sindemias, es decir, la presencia concurrente de varios problemas de salud ^1^, así como de efectos sociales, económicos y políticos de diversa índole ^2^. Una visión simplista ha centrado la discusión en si se debe priorizar la salud o la economía de las sociedades ^3^, pero la problemática es compleja y hay que intentar minimizar ambos efectos dado que en el corto, mediano y largo plazo están íntimamente relacionados con consecuencias negativas en lo económico y en lo sanitario.

Aunque se sabe que la mayoría de contagios ocurre en el hogar ^4^, por las condiciones de intimidad y cercanía de los miembros de las familias, el sitio de trabajo también es un lugar donde debe mitigarse la pandemia sin detener la producción. En ese sentido, las empresas pueden convertirse en sitio de diagnóstico de la infección, de evaluación de la respuesta inmunológica y de verificación del arribo a la inmunidad de rebaño, con lo que se completa el cuadro de la vigilancia basada en los casos confirmados, que constituyen solo la punta del iceberg de la epidemia ^5^. Dado que todos estos elementos son fundamentales para la adecuada planeación empresarial, una opción razonable es propiciar la continuación de las actividades económicas con bioseguridad y vigilancia ocupacional adecuadas, de manera que la productividad se mantenga sin menoscabo de la salud de los trabajadores.

En tal contexto, este estudio presenta el desempeño de las pruebas combinadas de IgM e IgG rápidas en empresas colombianas de diferentes regiones que decidieron hacer frente a la pandemia reorganizando el trabajo y haciendo vigilancia ocupacional con pruebas serológicas masivas, las cuales se complementaron con pruebas de reacción en cadena de la polimerasa con transcriptasa inversa *(reverse transcriptase quantitative PCR,* RT-qPCR). Aunque los resultados aquí presentados son parciales (primeros tres meses), la estrategia puede implementarse en otras empresas para mantener la productividad y contribuir al manejo de la pandemia.

## Materiales y métodos

Se usaron los datos de la vigilancia ocupacional de empresas localizadas en diversas regiones del país que prefirieron mantener su anonimato y siguieron un programa de vigilancia de la infección con SARS-CoV2 en el marco de las actividades de salud ocupacional usando pruebas combinadas de IgM e IgG rápidas y una organización de los trabajadores en grupos pequeños como ejes de la estrategia. Entre una empresa y otra hubo las diferencias lógicas inherentes al sector económico y los procesos productivos específicos, aunque la mayoría de ellas están en la producción de alimentos o en actividades relacionadas con sus subproductos o con su logística. La estrategia empezó a implementarse en la segunda mitad de abril del 2020 cuando los casos de COVID-19 en Colombia se concentraban principalmente en Bogotá, Cali y el departamento de Antioquia ^6^. En ese momento, la capacidad de los laboratorios para hacer el diagnóstico de la infección de SARS-CoV2 era muy limitada, por lo que la vigilancia ocupacional se basaba en la detección de síntomas y en el uso de pruebas serológicas combinadas de IgM e IgG rápidas.

Los datos para los análisis se recolectaron de manera *ad hoc* según los lineamientos definidos por un comité empresarial creado para tal fin, el cual contaba con la participación de uno de los autores del presente estudio. Se partió de la premisa de que, por definición, los trabajadores son más sanos que las poblaciones de las que proceden ^7^, es decir, al llamado "efecto del trabajador sano" ^8^, por lo que *a priori* se sabía que la mayor parte de los trabajadores sería asintomática. Los trabajadores aceptaron los cambios organizacionales después de las actividades de información y comunicación de los riesgos asociados con la infección por SARS-CoV2.

### Estrategia de organización del trabajo

Siguiendo los protocolos y lineamientos gubernamentales (principalmente la Resolución 666 de 2020 del Ministerio de Salud y Protección Social), se establecieron y reforzaron diversas estrategias básicas de bioseguridad en el trabajo. Las más frecuentes incluyeron el uso adecuado y constante de tapabocas, el lavado periódico y correcto de las manos, no compartir objetos personales y de trabajo en lo posible, y diversas medidas de distanciamiento físico entre los trabajadores. La principal estrategia complementaria fue la reorganización de los trabajadores en grupos pequeños, independientes y aislados los unos de los otros, estrategia que puede considerarse una expresión de lo que posteriormente se ha conocido como "burbujas sociales" en la literatura científica ^9^. Para garantizar que el distanciamiento fuera real, se adoptaron medidas como el uso de colores en la indumentaria y otros elementos para identificar más fácilmente los miembros de los grupos y la delimitación de áreas permitidas y prohibidas para ellos, entre otras.

Las llamadas "burbujas sociales" se basan en redes conductuales que sirvieron de fundamento de tres estrategias de manejo del contagio con SARS-CoV2: el contacto con personas similares, el fortalecimiento del contacto en las comunidades, y la interacción repetida con las mismas personas dentro de una burbuja ^9^. Esta última fue la utilizada en las empresas participantes en este estudio y en ese marco los individuos decidieron con quiénes interactuarían regularmente, restringiendo su interacción social a dichas personas, con el fin de disminuir la probabilidad de infección y, en caso de que esta ocurriera, la transmisión a otras. De las tres estrategias basadas en redes sociales, la que ha resultado teóricamente más efectiva es la de "burbujas sociales" ^9^.

### Estrategia de vigilancia ocupacional

**Figura 1:**
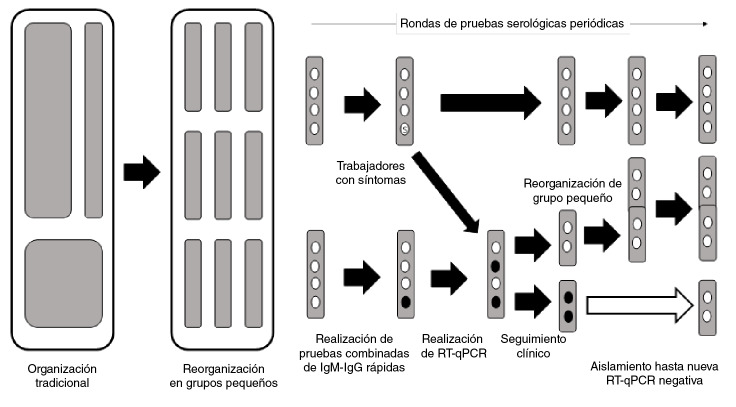
Esquema de reorganización del trabajo en grupos pequeños, pruebas serológicas periódicas y manejo de casos positivos en las empresas colombianas participantes

Todos los trabajadores con actividades presenciales en las empresas fueron incorporados a un programa de vigilancia ocupacional con pruebas serológicas periódicas combinadas de IgM e IgG rápidas ^10^ y encuestas diarias sobre los síntomas relacionados con la infección por SARS-CoV2. En la figura 1 se presenta un resumen de la estrategia de vigilancia ocupacional. Cuando uno o más trabajadores de un grupo pequeño resultaba positivo en una prueba [IgM(+)/IgG(-) o IgM(+)/IgG(+)], todos los trabajadores del grupo eran aislados en sus hogares y se les aplicaba una RT-qPCR. Quienes resultaban positivos en la prueba RT-qPCR quedaban aislados en sus domicilios hasta obtener resultados negativos de la prueba RT-qPCR y, una vez se cumplía esta condición, podían volver a incorporarse a otros grupos conformados especialmente por trabajadores temporales, y el proceso de vigilancia ocupacional continuaba. Las pruebas serológicas cumplían con los requisitos exigidos en Colombia ^11^ y con todos los lineamientos empresariales para la prevención de COVID-19, los cuales son más exigentes que las normas adoptadas por el gobierno.

### Evaluación de las pruebas combinadas de IgM e IgG rápidas

Dado que las pruebas se utilizaron en un contexto de vigilancia ocupacional, es decir, primero se aplicaban masivamente las pruebas rápidas y luego se complementaban con la RT-qPCR, no fue posible usar las fórmulas habituales para la evaluación de pruebas diagnósticas ^12^. El diseño de la vigilancia ocupacional se parece más al llamado diseño con muestreo prospectivo de evaluación de tecnologías diagnósticas, por lo que las características de las pruebas combinadas de IgM e IgG rápidas se verificaron mediante el ajuste de las probabilidades condicionales, tal como fue propuesto por Kraemer ^13^.

Explicado brevemente, dado que solo un subgrupo de los sometidos a las pruebas rápidas fue diagnosticado con la RT-qPCR, se recurrió a procedimientos bayesianos para estimar los verdaderos positivos (VP) y los falsos negativos (FN) en la muestra. Cada estimación se hizo a partir de quienes resultaron positivos dentro de la muestra sometida a la prueba rápida, es decir, el llamado nivel de la prueba (Q), así como el valor predictivo positivo (VPP) y el valor predictivo negativo (VPN) de los sometidos tanto a la prueba rápida como a la RT-qPCR. De esta manera, los VP son el resultado del producto de Q y el VPP, y los FN, del producto de (1-Q) y (1-VPN). Con estos datos fue posible estimar la prevalencia (P) como la suma de VP y FN, es decir: P = (Q x VPP) + [(1-Q) x (1-VPN)] y a partir de allí se estimó la sensibilidad (S) como el producto de Q y (VPP / P) y la especificidad como el producto de (1-Q) y [(VPN / (1-P)] ^12^.

### Evaluación de la respuesta inmunológica

La historia natural de la infección con SARS-CoV2 puede seguirse con la medición de la presencia del virus y la respuesta inmunológica del individuo infectado. Así es posible evidenciar que los estadios iniciales de la infección se detectan con la RT-qPCR, en tanto que con la IgM se constata la primera respuesta inmunitaria del individuo infectado, en tanto que la IgG se mantiene positiva hasta después de terminada la infección ^14^^-^^16^. Para fines ocupacionales, los trabajadores con lgM(-)/lgG(-) o lgG(+) son aptos para trabajar porque no presentan la infección, o ya la superaron; si los trabajadores están en los estadios de lgM(+) o lgM(+)/lgG(+), la infección está activa y podrían contagiar a los compañeros.

El conocimiento de la transición de la respuesta inmunitaria permite la planificación empresarial, pues determina el momento en que los trabajadores deben ser aislados, el tiempo que deben permanecer así y el momento en que pueden retornar a las actividades laborales sin convertirse en un posible foco de contagio para el resto de los trabajadores.

## Resultados

En el análisis se incluyeron datos de 4.234 trabajadores cuyas labores exigían su presencia en la empresa, es decir, cerca del 90 % del personal de las empresas participantes. Este número varió porque a los trabajadores con múltiples enfermedades o edad avanzada se les invitó a quedarse en sus hogares por prevención y se los reemplazó con trabajadores temporales. Los cálculos para obtener las características de las pruebas combinadas de IgM e IgG rápidas se encuentran en el cuadro 1. Como puede apreciarse, los resultados no muestran un desempeño óptimo, como el requerido en la práctica clínica. La sensibilidad y la especificidad de las pruebas IgM e IgM/ IgG fueron menores a las esperadas de este tipo de prueba en el proceso diagnóstico. Los valores predictivos negativos en ambos escenarios tuvieron un rendimiento de 18,55 y 4,3 %, respectivamente.

**Cuadro 1:** Cálculos de las características de las pruebas combinadas de IgM e IgG rápidas en trabajadores mayoritariamente asintomáticos

RT-qPCR*	IgM(+)		IgM(+) / IgG(+)	
	+ / n	%	+ / n	%
VPP	70 / 129	54,26	82 / 160	51,25
VPN	23 / 124	18,55	4 / 93	4,30
Q = (VP + FP)	(70 + 59) / 253	50,99	(82 + 78) / 253	63,24
Q' = 1 - Q	49,01		36,76	
P	67,59		67,59	
P' = 1 - P	32,41		32,41	
S	40,94		47,95	
E	28,05		4,88	

* Prueba de referencia

VPP: valor predictivo positivo; VPN: valor predictivo negativo; Q: nivel de la prueba; P: prevalencia; S: sensibilidad; E: especificidad

Sin embargo, dado que la detección de un trabajador positivo obligaba al aislamiento preventivo y a la confirmación con RT-qPCR, sí resultó ser una estrategia efectiva para evitar la presencia de brotes mediante medidas de aislamiento, sanitización y reorganización empresarial y, en algunos casos, la detención temporal de algunas operaciones de las empresas. Por cada trabajador positivo detectado con las pruebas combinadas de IgM e IgG rápidas [IgM(+) o IgM(+)/IgG(+)], y consecuentemente aislado, se aislaron preventivamente uno o dos compañeros de trabajo de su mismo grupo pequeño ("burbuja social") con IgM(-)/IgG(-). Dado que a todos los trabajadores del grupo pequeño se les hacía la RT-qPCR, se pudieron diagnosticar casos positivos que fueron notificados a las autoridades sanitarias cuando ya llevaban algunos días de aislamiento. De esta manera, además, se aumentó artificialmente la sensibilidad de las pruebas combinadas de IgM e IgG rápidas.

**Figura 2:**
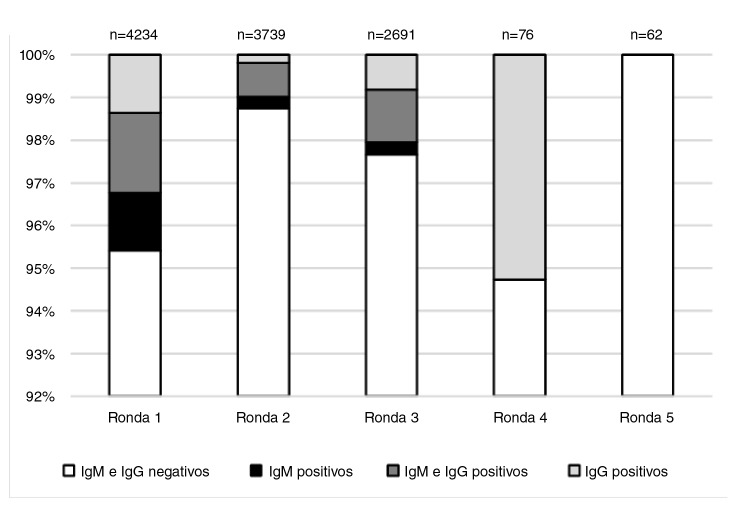
Cambios observados en las rondas de pruebas serológicas realizadas a los trabajadores de las empresas incluidas en el estudio

Los resultados indican que hubo 171 muestras con algún nivel de inmunoglobulina positiva y 82 muestras con resultados negativos. Del total de positivas, 67 tuvieron IgM(+), 82 tuvieron IgM(+)/IgG(+), y 70, IgG(+), como se observa en la figura 2. La IgG de un muy pequeño grupo de trabajadores evidenció que la infección ya había pasado sin haber tenido síntomas, lo cual ocurrió en los municipios donde la epidemia llegó con más fuerza y primero que a otros lugares.

## Discusión

Los hallazgos de este estudio indican que las características y rendimientos operativos de las pruebas fueron inferiores a los esperados para este tipo de pruebas, que suelen ser muy sensibles. Esto se explicaría porque, por un lado, en la mayoría de los lugares donde las empresas del estudio operan, la epidemia estaba en sus fases iniciales, lo que significaba que la prevalência de la enfermedad no era suficiente para un adecuado rendimiento de las pruebas ^17^ y, por otro, las características operativas de las pruebas deben considerarse en el momento de comprarlas, ya que su comportamiento en términos de detección de casos depende de ellas y del estado de la epidemia en el contexto local ^17^^,^^18^.

Si bien las características de las pruebas combinadas de IgM e IgG rápidas no se consideran adecuadas para el uso clínico individualizado, desde el punto de vista de la salud ocupacional y la vigilancia epidemiológica sí tienen utilidad. Nuestros hallazgos son similares a los reportados en estudios previos ^16^, en los que las pruebas serológicas rápidas tuvieron una baja sensibilidad y los falsos negativos constituyen un problema inherente. No debe olvidarse que en el contexto laboral se busca hacer la tamización entre los trabajadores asintomáticos para adoptar acciones preventivas poblacionales y no tanto para hacer diagnósticos clínicos individuales. En situaciones de crisis sanitaria, cuando los asintomáticos tienen un papel fundamental en la transmisión de la enfermedad, varios autores han propuesto aplicar pruebas rápidas seriadas cada dos o tres días en ellos para mejorar así el desempeño de la prueba en términos de la sensibilidad y la probabilidad de detección y, por lo tanto, disminuir la posible propagación del virus mediante medidas de aislamiento temprano, esto en contraposición con la PCR, la cual confirma la infección pero en momentos epidemiológicos tardíos (4 o 5 días después del inicio de los síntomas) ^19^^,^^20^.

La utilización de las pruebas combinadas de IgM e IgG rápidas en los grupos pequeños de trabajo ("burbujas sociales") permitió controlar, por lo menos parcialmente, la pandemia dentro de las empresas. Si no se hubiera implementado esta estrategia, no se habría podido conocer la infección de los trabajadores asintomáticos, con la consecuente incertidumbre y la imposibilidad de controlar la situación. Hasta donde se sabe, estos hallazgos son similares a los de los brotes ocurridos en empresas parecidas en varios lugares dei mundo. En este sentido, vale la pena señalar que el control parcial permitió definir, probar y cambiar estrategias rápida y oportunamente, ajustándolas a las diversas situaciones de la operación de las empresas.

**Figura 3:**
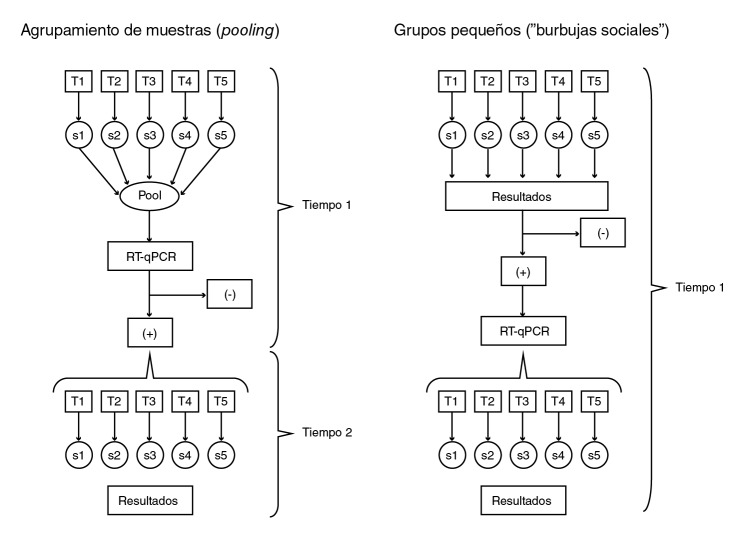
Comparación teórica entre el análisis de muestras mediante agrupación de muestras *(pooling)* y la organización de grupos pequeños ("burbujas sociales")

Esta forma de manejo se asemeja al agrupamiento de múltiples muestras biológicas conocido como agrupación de pruebas de diagnóstico *(pooling diagnostic tests),* el cual se basa en la propuesta de Dorfman hecha a mediados del siglo pasado para hacer más eficiente la inspección de grandes poblaciones ^21^, y cuyo uso en los diagnósticos de salud ha probado ser beneficioso, especialmente en situaciones de contagio rápido de enfermedades infecciosas, pues se agrupan varias muestras en una única para el análisis ^22^. En la figura 3 se comparan los agrupamientos de múltiples muestras diagnosticadas con la prueba de referencia y la estrategia de los grupos pequeños y se evidencia que el diagnóstico inicial con las pruebas rápidas fue de menor sensibilidad. Es evidente que el tiempo requerido con las pruebas rápidas es menor, lo que permite disminuir también el tiempo de aislamiento de los individuos y, lo que es más importante, la transmisión del contagio por SARS-CoV2. También es posible que, en algunas situaciones que dependen de la magnitud del evento, resulte ser menos costoso el diagnóstico de grandes poblaciones, por lo que el agrupamiento de muestras y el uso de grupos pequeños son estrategias que aumentan la eficiencia de las pruebas diagnósticas.

Este estudio presenta una experiencia exitosa de manejo de la pandemia de COVID-19 en un grupo de empresas privadas colombianas que, reconociendo el riesgo, decidieron organizar una estrategia en un contexto de poca disponibilidad de pruebas diagnósticas ^23^. Se sabe que las reuniones en lugares de culto religioso, los dormitorios empresariales, los hospicios para adultos mayores, los hospitales, los bares, las escuelas, los eventos deportivos masivos y los cruceros pueden ser la ocasión perfecta para los brotes ^4^, a ellos se suman las empresas procesadoras de alimentos (como varias de las incluidas en este estudio), pues sus trabajadores suelen laborar en proximidad con controles de temperatura para la manipulación de los alimentos ^24^.

En conclusión, la experiencia ocupacional aquí presentada es un ejemplo de cómo salvaguardar la salud de los trabajadores y mantener simultáneamente las actividades productivas. Es un claro esfuerzo de un grupo de empresas privadas para afrontar la pandemia que puede servir de modelo para otras que busquen ajustarse a la llamada "nueva normalidad" En futuros estudios se podrá evaluar el nivel de inmunidad de rebaño registrado al emplear pruebas combinadas de IgM e IgG rápidas ^25^^,^^26^, que en este no se hizo porque el momento de la epidemia en el país no estaba suficientemente avanzado para recabar los datos necesarios. A finales de septiembre de 2020, fecha de redacción de este manuscrito, ya se estaban empleando las pruebas de antígenos ^27^, las cuales tienen mejor sensibilidad y especificidad que las serológicas, por lo que las empresas rápidamente se adaptaron a esta nueva situación y las introdujeron en la vigilancia ocupacional.
